# Technology-Enabled Care: Integrating Multidisciplinary Care in Parkinson's Disease Through Digital Technology

**DOI:** 10.3389/fneur.2020.575975

**Published:** 2020-10-30

**Authors:** Raquel Luis-Martínez, Mariana H. G. Monje, Angelo Antonini, Álvaro Sánchez-Ferro, Tiago A. Mestre

**Affiliations:** ^1^Department of Neurosciences, University of Basque Country (UPV/EHU), Leioa, Spain; ^2^Department of Neurosciences (DNS), Padova University, Padova, Italy; ^3^HM CINAC, Hospital Universitario HM Puerta del Sur, Universidad CEU-San Pablo, Madrid, Spain; ^4^Division of Neurology, Department of Medicine, The Ottawa Hospital Research Institute, Parkinson's Disease and Movement Disorders Center, The University of Ottawa Brain Research Institute, Ottawa, ON, Canada

**Keywords:** Parkinson's disease, technology, multidisciplinary care model, home care (HC), rehabilitation

## Abstract

Parkinson's disease (PD) management requires the involvement of movement disorders experts, other medical specialists, and allied health professionals. Traditionally, multispecialty care has been implemented in the form of a multidisciplinary center, with an inconsistent clinical benefit and health economic impact. With the current capabilities of digital technologies, multispecialty care can be reshaped to reach a broader community of people with PD in their home and community. Digital technologies have the potential to connect patients with the care team beyond the traditional sparse clinical visit, fostering care continuity and accessibility. For example, video conferencing systems can enable the remote delivery of multispecialty care. With big data analyses, wearable and non-wearable technologies using artificial intelligence can enable the remote assessment of patients' conditions in their natural home environment, promoting a more comprehensive clinical evaluation and empowering patients to monitor their disease. These advances have been defined as technology-enabled care (TEC). We present examples of TEC under development and describe the potential challenges to achieve a full integration of technology to address complex care needs in PD.

## Introduction

Parkinson's disease (PD) is a neurodegenerative disorder with motor and non-motor clinical manifestations (NMS) that dictate the accrual of loss of autonomy and increasing complexity of care. The increase in life expectancy and expected doubling of PD prevalence in coming years (1) further support the development of PD management strategies with high dissemination and greater usability potential.

The organization of healthcare teams dedicated to care delivery for people living with PD (PwP) is an active research field. The vast majority of system-based approaches consist of care delivery models centered in a PD tertiary center either in the form of an all-in-one multidisciplinary clinic or as a hub of a care network articulated with regional healthcare centers.

The use of technology in PD has gathered great interest. The potential to generate a more continuous and remote health monitoring and the enhancement of patient care communication are bound to deliver a revolution in PD care.

In this review, we first introduce concepts and state-of-the-art knowledge about the use of technology in PD evaluation, the approaches to multidisciplinary care, and the concept of technology-enabled care (TEC). We provide real-world scenarios on how these three concepts can be implemented jointly in a digital revolution for care today and in the future.

## Technology in PD: Overview and Core Concepts

In the last decades, there has been a growing interest in improving health-related outcomes using technology. In PD, technology-based solutions have been developed mainly with the aim of generating an accurate, objective, and reproducible measurement of motor function. Novel sensor-based and wearable technologies enable a shift of the evaluation of PD from the traditional clinical examination and clinical scales to one based on more objective health monitoring of daily function in an everyday-life naturalistic environment. For example, the detailed analyses of movement patterns in the home are expected to provide greater insight on patients' clinical status and their response to treatment.

The most relevant new technologies supporting this paradigm change are inertial measurement units (IMUs). Most IMUs have a triaxial accelerometer and gyroscope, although a magnetometer is frequently included. IMU-based devices are based on the same general principles: (a) preprocessing of the signal generated by the IMU, (b) extraction of the essential characteristics of the movement signal, and (c) creation of a summary variable of the pattern of movement (2). Other examples of technologies being used include virtual reality (VR)-based systems, optoelectronic systems, or a combination of these (3).

IMUs have been embedded in devices worn by the patient (i.e., wearable sensors and systems) in the clinic and, for remote monitoring, in the home setting. As such, wearable technology may more realistically portray motor function for clinical and research purposes. Currently, technologies developed for the management and treatment of PwP have enabled measurement of variations in movement parameters, such as frequency and amplitude that have moderate to high agreement with traditional motor standards such as the Movement Disorders Society-Unified Parkinson Disease Rating Scale (MDS-UPDRS) (3, 4). These data could potentially allow clinicians to assess the full spectrum of PD's clinical manifestation including the presence and severity of the cardinal features and treatment-related motor complications of PD (4). Less frequently, technology may be used to monitor NMS such as cognition, sleep, dysautonomia, and neuropsychiatric features (3). The main challenges to the mature development of these technologies include the ability to capture the full spectrum of the disease, standardize validation protocols, use naturalistic environments to determine ecological validity, and enhance the maturation processes of assessment systems with a particular focus on the definition of the context of clinical use from early stages of development (5).

Ultimately, the development of sensor-based and wearable technologies and the growing internet-enabled access to information and mass data storage would facilitate the integration of these technologies in a multisensor/multidomain healthcare framework that we describe below (see the Technology-Enabled Care section).

## Models of Multispecialty Care

Currently, allied health interventions are carried out most commonly in isolation, with insufficient collaboration and communication with other disciplines involved in PD care (6, 7). The actions of a broad group of physicians and other healthcare professionals in PD care warrant a dedicated organization to optimize care delivery to PwP. The different approaches to multispecialty care can be broadly divided into three categories. (i) In multidisciplinary care, each care provider is responsible for a specific patient care need in the absence of standardized coordination. Commonly, the care providers in this care model are colocated in a single location, raising issues of feasibility and wide dissemination for providing a holistic care for PwP. (ii) In interdisciplinary care, there is active collaboration of healthcare team members to make group decisions. (iii) In integrated care, a care plan is delivered by a coordinated team of healthcare providers (2) guided by consensus building and engagement of patients as team members (3, 4). Integrated care involves the support to the navigation of care resources available in the hospital and community and, more commonly, includes patient education and self-management combined with a structured clinical follow-up and case management. Initial evaluations of integrated care delivered as a PD-dedicated care network in the community with a specialized PD nurse playing the role of a care integrator documented an improvement in quality of life (QoL) and patient and caregiver satisfaction over 6 months (6, 7).

## Technology-Enabled Care

Technology can play a significant role in care delivery in PD as it is designed to increase the engagement of people in their healthcare and foster self-management in a highly personalized way. The term TEC has been adopted to express the transformative potential of different technological solutions such as telemedicine, online coaching, and self-care apps for care. TEC aims to cover the following goals in the PD care paradigm: (i) assess and measure a wide range of symptoms to capture subtle changes at the prodromal stage and document clinical progression, (ii) support therapeutic choices especially in the presence of multimorbidity, (iii) facilitate rehabilitation and physical activity, and (iv) facilitate remote care.

There are two critical gaps in the care of PwP that technology can help overcome. First, most commonly, each specialty provides care in a silo. Second, with few exceptions, the patient's current assessment is restricted to the hospital or clinic setting. Three main technological breakthroughs can enable care integration supported by technology. One is the digitalization of medicine, which permits patients' connectivity with the hospital from the home environment and the connection between specialists (8); second is the availability of wearable devices that can objectively monitor the patient outside of the hospital/outpatient environment as described before. Finally, technologies for neurorehabilitation are also enabling some models of care in the home setting.

**Figure 1:**
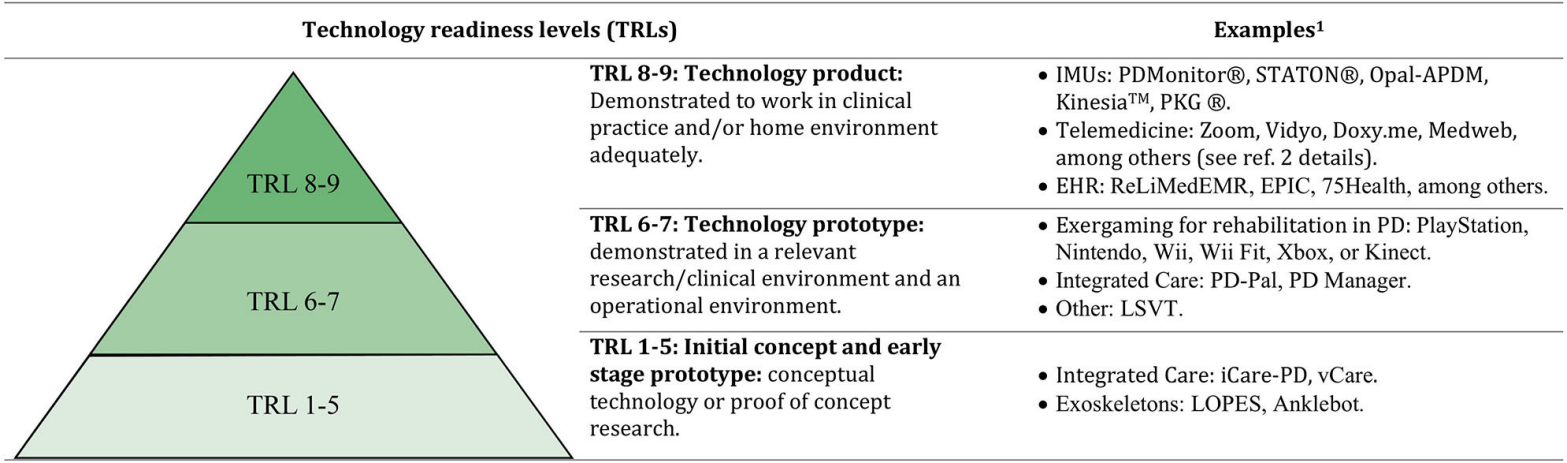
Technology readiness levels (TRLs) and systems mentioned in the review. IMUs, Inertial Measurement Units; EHS, Electronic Health Records; LSVT, Lee Silverman Voice Treatment Device; LOPES, Lokomat, ReoAmbulator, Lower Extremity Powered ExoSkeleton. ^1^Based on two authors' (MHGM, ASF) consenus opinion after reviewing available literature as of June 23, 2020. ^2^https://www.movementdisorders.org/MDS/About/Committess––Other-Groups/Telemedicine-in-Your-Movement-Disorders-Practice-A-Step-by-Step-Guide/Step-1-Obtain-Necessary-Equipement-and-Software-Equipement.htm.

An important aspect to highlight is that not all systems bear the same degree of development. Like drug trials, where the different phases reflect how close a new drug is from being approved for medical use, in technology, the maturity or “readiness level” reflects how close a system is to being validated for use in routine care. The Technology Readiness Level (TRL) scale developed by NASA in the 1970s is a scale commonly used for this purpose (9) (Figure 1). We will review the status of the different technological breakthroughs introduced here.

### Digital Health and the Connectivity of Patients and Specialists

Digital health technologies, namely, telemedicine, telehealth, and health information technologies, have the potential to reduce the burden of care by connecting patients with the specialist and deliver personalized health services directly to the home (10), supporting multidisciplinary care to manage the complex care needs of PwP (7). Multiple online digital health platforms are available and have a TRL9 (Figure 1) for connectivity between patients and clinicians (10). Web-based video conferencing solutions may offer similar clinical benefits to in-person care, while saving patients and caregivers an average of 100 miles of travel and 3 h compared with regular in-person visits (11). In addition, digital heath initiatives suggest that comprehensive PD home-based care models are feasible and have the potential to integrate multispecialty data and care (e.g., physiotherapy, speech therapy, and telerehabilitation). The most advanced initiative is the ParkinsonNet, a multidisciplinary care model in the Netherlands. In this network, remotely supervised home-based aerobic exercise was feasible and had a positive impact on the motor aspects of PD (12). Despite their proven added value, current online platforms do not provide integration and real-time communication among different care providers and have a low technological maturity (TRL2) for this specific use.

Digitalization is also characterized by the progressive use of electronic health records (EHRs), which in the last decade has been an essential advance for the efficient transformation of medical care institutions. EHRs have proven essential for preventing medical errors, improving efficiency and quality, increasing costumers' trust, improving medical care, and cutting down on healthcare costs (13). Electronic repositories can overcome the ineffectiveness of traditional paper-based records, usually used to store and organize an ever-increasing number of diverse data. EHRs enable the complete integration of PwP health status across providers, generating an interactive and flexible platform to communicate. For instance, Epic Systems Corporation (EPIC), iPatientCare EHR, ReLi Med Solutions (ReLiMedEMR), or 75Health proposes a software solution to support patient care, namely, patient registration, visit scheduling, and medical staff access.

The SARS-CoV2 pandemic has amplified the need to adopt digital healthcare (14). Both health professionals and patients demand technologies that enable integrated multispecialty care beyond the hospital and facilitate knowledge exchange among professionals, a concept called “liquid hospitals” by some (15). Despite this need, its implementation is challenging (16). Other barriers worth mentioning are internet access, preservation of privacy, and data protection. In summary, the digitalization of medicine positions itself as the main driver of TEC, once the integration between different specialists can be widely used securely and privately.

### Sensor Technology

Another key element of TEC is the sensing of different health-related phenomena at “home,” more specifically, the natural environment of the patient. Wearable devices enable the remote assessment of patients' conditions in their natural settings (17) and measure relevant outcomes (e.g., physical activity, sleep, and falls), which are hard to assess in a regular outpatient clinical visit using clinical interview, patient recall, and clinical exam time-locked to a given visit.

**Figure 2:**
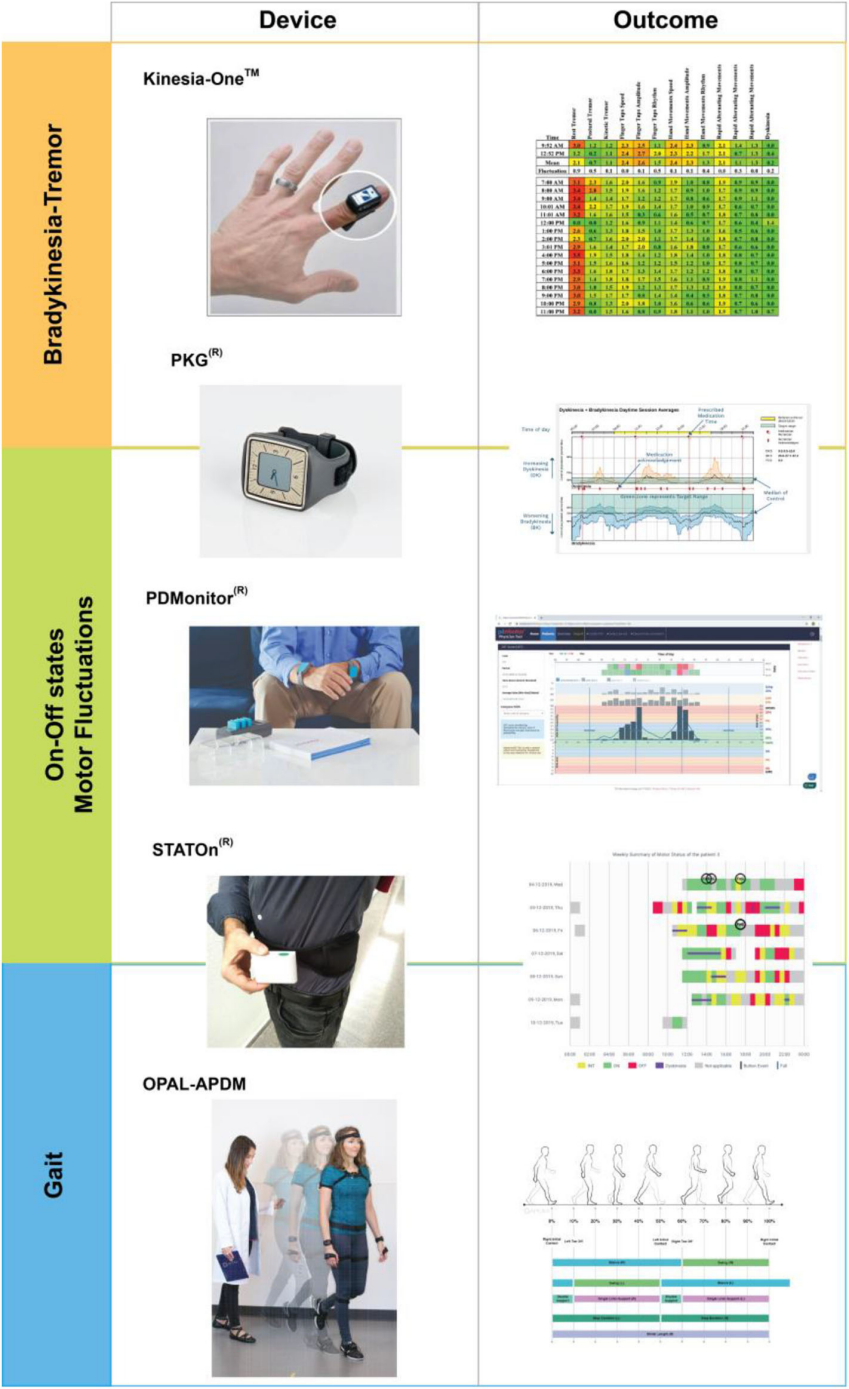
Inertial measurement unit devices approved for used in movement disorders. Relevant examples of different devices and the generated information are presented according to their main application (i.e., the Kinesia system can also be used for dyskinesia and gait, see Table 1). The different results obtained with the use of each of the devices are presented. Images provided by Kinesia™, Great Lakes NeuroTechnologies; PKG®, Global Kinetics Corporation; PDMonitor®, PD Neurotechnology® Medical Solutions; STAT-ON, Sense4Care; Opal, APDM Wearable Technologies. Adapted from *Monje MHG and Sánchez-Ferro A. Sistemas inerciales y análisis del movimiento. In: Manual de Nuevas Tecnolog*í*as en Trastornos de Movimiento, 2020 (in press)*.

As mentioned earlier, IMUs represent the most widely used technology used in PD and may well-serve the goal of providing data meaningful for healthcare. Over time, IMU-based sensors have become more refined and portable, allowing for unobtrusive monitoring of PD in the home environment. Currently, the main applications of these sensors include (i) the accurate evaluation of cardinal motor features (mainly for bradykinesia and tremor) (18, 19) and (ii) the detection of complications that appear throughout the disease (e.g., the exact quantification of on vs. off states and motor fluctuations or the freezing of gait and falls in a home environment) (20, 21) (Figure 2). For example, the Kinesia™ system uses an IMU placed on the patient's index finger or the heel and can differentiate between a healthy subject and a patient with bradykinesia and measure the presence of tremor (18, 26). Other systems like the PDMonitor® (multisensors), the PKG® (clock-shaped IMU), or Mobility Lab System-APDM® can continually record several motor signals and differentiate between motor patterns, on–off states, and dyskinesia (19, 22, 23). On the other hand, other devices can detect movement transition changes (e.g., falls and posture transitions). Significantly, the STAT-ON® device, a waist position device, can detect motor fluctuations (on–off periods) for PD advanced stages or even freezing of gait, which is potentially groundbreaking progress for PD management (24). Currently, the above-mentioned devices created for the evaluation of PwP have reached the maximum level of development (i.e., TRL9) (Table 1) and have been approved by regulatory agencies in the EU and USA for routine clinical practice (e.g., for the remote monitoring of axial motor symptoms, bradykinesia, and tremor) (18, 20, 22, 27). Other systems using other types of sensors or tailored to detect other manifestations have a lower TRL (3).

**Table 1:** Currently available systems with advance regulatory status for the objective quantification of movement in Parkinson's disease patients.

**System**	**Application**	**Use**	**Performance**	**Sensor**	**Outcome**	Regulatory status^*^
Kinesia-ONE™ Kinesia-360 (18)	Tremor Bradykinesia Dyskinesia	Clinical practice Home Research	MDS-UPDRS III tasks	- Distal index finger - Heel	MDS-UPDRS-based score (0 to 4)	CE mark FDA approved
Personal KinetiGraph® (PKG) (19)	Bradykinesia Dyskinesia Gait (continuous monitoring)	Clinical practice Home Research	Free activity	-Wrist	Time in ON–OFF, time with dyskinesia	CE mark FDA approved
PDMonitor® (22)	Bradykinesia Dyskinesia (continuous monitoring)	Clinical practice Home Research	Free activity	-Both wrists -Both feet	Time in ON–OFF, time with dyskinesia, freezing of gait, falls	CE mark
Mobility lab system-APDM® (23)	Gait (continuous monitoring)	Clinical practice Research	TUG Free activity	-Both wrists -Both feet -Waist	Gait parameters (speed, cadence, swing)	CE mark FDA approved
STAT-ON (24)	Gait (continuous monitoring)	Clinical practice Home Research	Free activity	-Waist	Duration of ON and OFF, freezing of gait, falls	CE mark
MoveMonitor-McRoberts (25)	Gait (continuous monitoring)	Clinical practice Home Research	TUG Free activity	-Waist	Type of activity and time in each activity	CE mark FDA approved

* *As listed in the respective companies' website or grey literature. The indication of use for each device as per CE Mark/FDA approval is linked to a specific clinical indication. Off-label use is not recommended. CE, Conformité Européenne; FDA, Food and Drug Administration; MDS-UPDRS-III, Movement Disorders Society-Sponsored Revision of the Unified Parkinson's Disease Rating Scale; TUG, Time Up and Go*.

The collection of wearable sensor data at home requires increased computing power, mass data storage capacities, and widespread internet access, which imply that the digitalization of medicine is enabled. The integration of multiple devices within the home environment may have a two-fold impact, allowing for a more comprehensive clinical assessment and empowering patients to monitor their disease in a delivery of highly personalized care (5). In the near future, we may witness the use of different sensors for a more comprehensive remote evaluation. Current technology-based gaps and challenges have been described elsewhere. The main barriers for TEC include the lack of integration among different wearable systems, the lack of consensus on patient-centered digital outcomes, and easiness to adopt technology (5). It is vital that standards of validation for these devices are widely used to overcome these barriers. Together with digitalization and connectivity, the expanding capabilities of sensors will allow movement of care from the hospital to the home in an integrated manner.

### Technologies for Neurorehabilitation

The field of neurorehabilitation is an ideal example of how technology could be implemented to support medical care. VR and augmented reality (AR) have become more popular recently in this field to enable remote care. A virtual environment established by a computer is used in VR, while in AR, the experience of a real environment is enhanced by computer-generated perceptual information. Since 2008, VR research in PD has been conducted with the first studies of gait evaluation using VR (28) Another more recent example is the use of smart glasses in PwP (29). Other studies have suggested that training in fully immersive VR can improve motor function, balance and coordination, cognitive function and mental health, QoL, and activities of daily living (30). Furthermore, VR offers the possibility of replicating real-life scenarios and may improve the effect of conventional rehabilitation therapy with a better performance in some PD manifestations, especially in balance and gait parameters (31, 32). However, more rigorously designed studies are necessary to provide stronger evidence.

In addition, commercial video games (VGs) like video games, exergames, serious gaming, PlayStation, Nintendo, Wii, Wii Fit, Xbox, or Kinect have shown positive results in combination with traditional physical therapy. VGs seem to be effective for treating gait, balance, and strength PD symptoms (33). Neurorehabilitation by exergaming has been confirmed as safe and flexible, has high adherence rates, and may enhance cognitive performance (34). However, due to the large variability in the protocols used (e.g., intervention of duration and number of sessions), studies linking game parameters with conventional assessments methods, such as MDS-UPDRS scores, are required. Likewise, insights into task-oriented exercises for transferring VG rehabilitation goals to real-life functionality are needed (33, 34).

VGs let patients interact in a two-dimensional environment real time and may represent a strategy to engage both mental and motor functions at the same time, possibly enhancing several PD cognitive domains (35). Exergames could be considered either as a supplemental treatment to conventional rehabilitation or as a strategy to extend the benefits of conventional programs at home (36).

Apart from that of cognitive functions, technological implementation for rehabilitation of other clinical manifestations such as speech and language is unfortunately limited. However, communication and swallowing problems, together with hypomimia, are highly prevalent in PD (37). A limited number of studies with the Lee Silverman Voice Treatment showed benefits on swallowing and reduced parkinsonian hypomimia (38). Maintenance of functional communication and swallowing over time is a considerable challenge for PwP, and more technological solutions are urgently required.

Exoskeletons and robotic devices are one of the technological advances in the field of neurorehabilitation. To date, several systems have been developed like the Lokomat, ReoAmbulator, Lower Extremity Powered ExoSkeleton (LOPES), and Anklebot (39). Although more data are required, some benefits have been found. Robotic-assisted gait seems to play a significant role in improving gait function and reducing freezing-of-gait episodes in PD (39–41), but the complexity and high costs of this multimodal integration must be carefully considered. In addition, the quality of evidence of current literature remains low. The studies are chiefly case reports (41).

## Integration of Technology in PD Care: Potential, Challenges, and Future Outlook

All the technologies described in the previous section and others not described here have the potential to reformulate PD management routines. Current standards for PD clinical care rely on assessment using clinical scales such as the MDS-UPDRS, Hoehn and Yahr staging, the Schwab and England rating of activities of daily living, and self-reported patient diaries (42). Although these are the most widely used scales in research and clinical routine, there are significant limitations. First, PwP often do not easily recognize motor features like dyskinesia, tremor, or motor fluctuations to fill in their diaries (43). Second, NMS like cognitive dysfunction, dysautonomia, fatigue, and pain contribute significantly to frailty and worsen QoL but are frequently underdiagnosed. To date, only a few comprehensive global scales are available, such as the Scales for Outcomes in Parkinson's disease and the Movement Disorder Society Non-motor Rating Scale (44). Moreover, the clinimetric limitations of clinical scales may lead to suboptimal measurement of motor symptoms and NMS, which in turn can negatively impact the provision of care (45). In the last decade, there has been growing interest in measuring health-related outcomes using technological devices and in the validation of digital endpoints. Therefore, many studies have investigated the characteristic manifestations of PD using technology-based devices, addressing a gap in the ability to monitor PD features over a long period. Technology objective measures in PD have been considered the cutting edge of unbiased measurements but remain yet to fully prove their clinical utility.

Traditional models of care focused on the management of a single chronic condition do not fit the paradigm of care required for PwP characterized by multimorbidity and frailty. In most healthcare systems, the “interface” between inpatient and outpatient management remains unsatisfactory and fragmented, which often leads to PwP receiving suboptimal care. Although elderly PwP will have other chronic diseases, most clinical guidelines focus almost exclusively on motor manifestations and neglect clinical heterogeneity (46, 47). Only recently have clinicians started to consider stratifying PwP based on progression of their functional disability, a process that may benefit from more profound integration of technology in routine care (48).

Wearable sensors, accelerometers, gyroscopes, and non-wearable devices have been tested as ambulatory devices to assess motor parameters such as gait, kinematic features, sway, physical activity, tremor, and bradykinesia (49, 50). These technologies can result in safe, objective, real-time behavioral assessments in clinical routine and facilitate the identification of care problems with more time dedicated to developing management plans and provide patient education during a clinical encounter. NMS have been less amenable to gyroscopic or accelerometer analysis in spite of their prevalence and significance for PwP. Albeit many aspects of cognition may be effectively monitored through neurocognitive tests applications, mood disorders are still complex to tackle. Simple technological approaches have failed in successful remote monitoring of anxiety or depression. In case of monitoring of sleep quality, biometric and sleep actigraphy monitors are already commercially available. In connection, sleep studies employed polysomnography and actigraphy to evaluate the quality of sleep in PD (51, 52) or even to diagnose PD-associated sleep conditions. For assessing large body movement during sleep, accelerometers have also been employed in several studies; however, the results have not been tied to any sleep quality. On the other hand, autonomic dysfunction remains underrecognized in PD (53), in part because its confirmation relies on cardiovascular autonomic testing available only in a few specialized laboratories (54). Overall, NMS technological development is imperative.

There are significant challenges in the implementation of technology objective measures in day-to-day clinical practice. PD is a progressive disorder, with a significant compromise of functional independence, self-care, and QoL. Moreover, it is frequently associated with multimorbidity, requiring a considerable number of clinical visits and hospital care, resulting in high medical and economic burden (55–57). The integration of technology in PD care needs to be safe, effective, patient-centered, timely, efficient, equitable, and secure. Several barriers exist for the appropriate clinical validation of available devices. Robust accuracy and validity in metrics are necessary with a high degree of confidence. The definition of compliance and feasibility for users is of particular relevance. In the absence of a proper definition and validation of TEC utility, the lack of accuracy, sensitivity, and reproducibility standards may lead to heterogeneous implementation and usage. Therefore, a future key development of healthcare technology is the need to create standard definitions using a multidisciplinary approach. Moreover, financial issues and universal technology access are also delaying the migration of care to the home. Thus, it is time to take this technological chance and face this challenge (5, 58–60).

The current technological development offers the opportunity to achieve an eHealth environment, where gaps of current care models are overcome and a more effective model of care is established. The foundational steps include implementing patient-operated digital platforms integrated with sensors and clinical and non-clinical applications, information sharing (e.g., health monitoring data, visit scheduling and timeline, and educational material) among patients and caregivers and healthcare providers, to complement face-to-face visits and enhance standard care pathways. The design of this technology needs to ensure engagement and effective use in real life. Suitable systems will be defined and used to support sensible and appropriate healthcare usage going beyond the traditional “telehealth” approach. The objective is to develop a system where multidisciplinary care managers and empowered patients operate and enable timely and coordinated access to healthcare providers.

## Integration of Technology in PD Care: Real-Life Examples

Thanks to the advances described above, new models of care delivery in PD begin to emerge, profiting from the advances in telecommunications (and technology at large), that enable the emerging generation of digitalization of medicine at “home.” Yet few of these models are integrated widely into PD management. Emerging care modalities require the unification of multispecialty teams and the migration of patient necessities into their home or community. In this context, PD-Pal, a multicenter European medical project, proposes an innovative approach to the care and management of PwP in the most advanced stages. At this stage, symptoms are complex, and treatment is challenging, with a severe compromise of QoL of patients and family members. Moreover, in this advanced PD stage, patient care necessities change frequently, which makes management difficult and leads to a high number of clinical visits. By integrating electronic tools to monitor movement and cognitive functions at home, for example, and defining the standards for an integrated multidisciplinary path, it will be possible to validate this approach. To achieve this goal, the project will incorporate the integration of a new wearable technology system, PDMonitor®, for remote patient monitoring in their natural environments. This device will inform the management of advanced PD patients overcoming architectural barriers and social isolation. The PD-Pal project could successfully shape multidisciplinary palliative care in PD, integrating technology at home and defining new European standards for care pathways in the advanced stages of PD (61).

The multinational consortium iCARE-PD is another example of technology integration for care delivery. iCARE-PD aims to develop an innovative, pragmatic healthcare model that shifts the hub of care from outpatient care to home-based community across a wider spectrum of disease stages in PD. This model consists of an integrated care network supported by a digital platform shaped as a virtual PD coach that incorporates principles of integrated care, self-management support, and TEC and integrates various eHealth solutions for PwP using co-design (62, 63). Co-design incorporates the input of stakeholders, namely, patients, care partners, and healthcare providers, in the development of technological solutions. The co-design in iCARE-PD is expected to enhance a patient-centered care delivery and, ultimately, to increase usability. Another aspect that characterizes the development of the virtual PD coach is the use of an agnostic platform. This feature will help to address the challenges of a hyperdynamic development of new technological solutions as it allows by design for any TOM to be incorporated at any time as a module of the virtual PD coach.

Another example is the vCare European project. vCare stands for virtual coaching activities for rehabilitation in the elderly and aims at improving rehabilitation for people as they age. vCare will develop and validate new information and communications technology based on a virtual coaching approach for empowering and motivating people with chronic diseases like PD. vCare proposes to support the recovery to an active and independent life at home, providing rehabilitation guidance and guaranteeing the continuity of care in the home environment. This project has the following aims: (i) coaching activities based on the underlying care pathway system; (ii) integration of a semantic layer enabling technologies such as reasoning, machine learning, behavioral models, and predictive analytics; and (iii) a continuous personalization regarding the cognitive, physical, and social conditions with seamless context integration and non-obtrusiveness in a home environment using open platforms like FIWARE (64). Therefore, this system would allow integration of clinical pathways, allowing a patient-specific adjustment of the rehabilitation program. The coaching environment will provide configurable services to personalize the intensity, content, and requests for optimal engagement of the patient to the individual rehabilitation program. Adequate health promotion can lead to a long-term behavioral change of habits, which decreases the economic effects and the probability of a relapse. This is especially so in the case of chronic diseases. Thereby, it becomes also an essential supplement for direct contact with the clinical specialists (65).

A final example of integrative PD management is a stand-alone technological integrated solution, the PD-manager. The PD-manager uses a set of mobile and wearable devices such as a smartwatch, smartphone, and sensor insoles for monitoring and collection of adherence data. The core of the system is a cloud system that provides all the necessary functionality for users and services communication, along with computing power for data processing and storage. This mHealth platform is accessible through the patients' mobile application and can be shared to clinicians to perform a clinical evaluation using a dedicated medical mobile application. Among the functionalities of the PD-manager, there is a pillbox to optimize medication intake, a dedicated nutritional study, game-based physiotherapy at home, and personalized management suggestions through education.

## Concluding Remarks

The current landscape of technology applied to PD evaluation and care is full of potential. The integration of technology in PD care is not a matter of possibility but how to fulfill the promise. For a successful implementation of TEC, it is urgent to create standards of validation for the intended clinical use of each technological modality and for their integration in a manner that is usable by patients. Ongoing and future collaborative projects will inform how the future eHealth environment will emerge to reduce care inequities and provide a more comprehensive care for empowered patients.

## Author Contributions

TM, AA, and Á-SF developed the structure and topic of revision. TM provided critique and review to the initial draft. All authors contributed to the initial draft.

## Conflict of Interest

The authors declare that the research was conducted in the absence of any commercial or financial relationships that could be construed as a potential conflict of interest.
